# Misdiagnosing Melioidosis

**DOI:** 10.3201/eid1302.061290

**Published:** 2007-02

**Authors:** Andrew J. Brent, Philippa C. Matthews, David A. Dance, Tyrone L. Pitt, Rupert Handy

**Affiliations:** * John Radcliffe Hospital, Headley Way, Headington, Oxford, United Kingdom; † Health Protection Agency (South West), Plymouth, Devon, United Kingdom; ‡ Laboratory of HealthCare Associated Infection, London, United Kingdom; § Heatherwood and Wexham Park Hospitals, Wexham, Slough, United Kingdom

**Keywords:** Melioidosis, Burkholderia pseudomallei, diagnosis, serology, letter

**To the Editor:** Melioidosis is endemic in southern and Southeast Asia and northern Australia. Although relatively few indigenous cases are recognized in the Indian subcontinent, a substantial proportion of cases imported into the United Kingdom originate there, probably reflecting patterns of immigration and travel, and underdiagnosis within the Indian subcontinent (*1*–*3*).

A 33-year-old woman spent 3 months in India. Shortly after arriving there, fever, myalgia, rigors, pharyngitis, and tender cervical lymphadenopathy developed. After she received antimicrobial agents, her symptoms initially improved, but in September 2005, 1 week after returning to the United Kingdom, she visited her general practitioner with recurrent fever and increasingly painful cervical lymphadenopathy. She was given a course of oral co-amoxiclav 625 mg 3× daily. However, the following week she visited the emergency department of her local hospital with left-sided suppurative cervical lymphadenitis. Pus aspirated from the lymph node grew an aminoglycoside-resistant “pseudomonad” identified as *Pseudomonas fluorescens* (API20NE profile 1056554), assumed to be a contaminant. She was discharged home to complete a further 10-day course of co-amoxiclav.

One month later, the patient again went to the emergency department, this time with a submental abscess. An otolaryngology consultation was sought, and the abscess was incised and drained. Although tuberculosis was suspected, no acid-fast bacilli were identified, and cultures were negative for mycobacteria; histologic examination showed noncaseating granulomata. Culture of fluid from the submental collection again yielded an aminoglycoside-resistant pseudomonad, however. At this point misidentification was suspected, and the isolate, which had a characteristic colony form on Ashdown’s Medium, microscopic appearance (Figure panel A), API20NE profile (1556574), and fatty acid profile, was identified as *Burkholderia pseudomallei*, the etiologic agent of melioidosis.

The patient had no relevant past medical history. Before immigrating to the United Kingdom 3 years earlier, she had lived in Tanjore, a rice-farming area of Tamil Nadu. She had stayed with family there during her recent trip, which coincided with the monsoon season, but she denied rural travel, fresh water contact, or skin abrasions. On examination, she was obese with acanthosis nigricans and tender cervical lymphadenopathy. Blood tests showed a mild microcytosis, low ferritin level, and erythrocyte sedimentation rate 40 mm/h; serum biochemistry and levels of C-reactive protein, fasting glucose, and hemoglobin by electrophoresis were normal. Two blood cultures were negative. Results of chest and abdominal imaging were normal. The patient was treated with intravenous ceftazidime for 10 days and oral co-trimoxazole for 4 months. She remains well.

*B. pseudomallei* serologic tests, performed subsequently, showed negative results by ELISA against the standard laboratory strain (204). However, when the assay was repeated using the patient’s own isolate, the result was positive (immunoglobulin G titer 4,000). Comparison of lipopolysaccharide (LPS) antigens from the 2 strains by sodium dodecyl sulfate–polyacrylamide gel electrophoresis and immunoblotting showed that they differed in O-repeating units (Figure panel B).

*B. pseudomallei* is an aerobic, gram-negative, environmental saprophyte ubiquitous in soil and surface water (e.g., paddy fields) in disease-endemic areas. Acquisition may occur through skin abrasions, aspiration of fresh water, inhalation, and possibly ingestion and may occasionally occur in the laboratory. An association between severe respiratory melioidosis and heavy monsoonal rains suggests that inhalation has previously been underrecognized as a route of infection (*4*); this is the likely mode in this case.

Many infections are initially subclinical but may result in latency and delayed manifestations, even after several decades. Clinical signs and symptoms include septicemia, cavitating pneumonia, bone and soft tissue infections, disseminated abscesses, mycotic aneurysms, lymphadenitis, and childhood parotitis. Most patients have an underlying predisposition to infection, especially diabetes, renal disease, alcoholism, and thalassemia, but in the largest Indian case series 50% patients had no traditional risk factors, as with our patient (*5*). *B. pseudomallei* is a category B potential bioterrorism agent.

Limited awareness of the disease, confusion with other conditions such as tuberculosis, and laboratory constraints all probably contribute to underdiagnosis of melioidosis in many areas (*6*). However, accurate diagnosis is important because septicemic melioidosis may be rapidly fatal, *B. pseudomallei* is intrinsically resistant to many antimicrobial agents, and prolonged treatment is usually required to minimize relapse. Diagnosis is usually by culture from sterile sites. Laboratory misidentification is not uncommon and occurred in this case because the diagnosis was not considered. Isolation of aminoglycoside-resistant pseudomonads in patients from disease-endemic areas should always prompt consideration of melioidosis and accurate identification. PCR is an emerging diagnostic tool not yet extensively validated (*7*).

The role of serology in diagnosis is limited by high background seropositivity rates in disease-endemic areas. No standardized serologic test is internationally agreed upon. This case illustrates another potential pitfall in melioidosis serodiagnosis. Most isolates express a conserved LPS antigen, which allows use of a single reference strain for determination of anti-LPS antibodies (*8*). However, because some strains express different LPS antigens, serologic tests must be performed with the patient’s own strain.

This case illustrates potential pitfalls in diagnosing melioidosis, which requires clinical and laboratory awareness and knowledge of its geographic distribution. LPS-based serologic assays should use a range of isolates representative of known LPS types.

**Figure:**
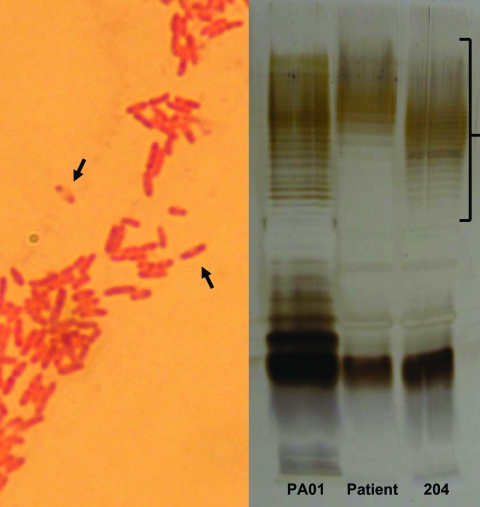
A) Gram stain of pus from the patient’s submental collection, showing the characteristic safety-pin pattern (arrows) of bipolar staining. B) Sodium dodecyl sulfate–polyacrylamide gel electrophoresis of lipopolysaccharide (LPS) antigens from the patient and *Burkholderia pseudomallei* reference strain (204), showing different O-repeating units (bracket). A control isolate of *Pseudomonas aeruginosa* LPS (PA01) is shown for comparison.
